# Optimizing Resin Infiltration Procedure in Molar Incisor Hypomineralization Lesions

**DOI:** 10.1111/jerd.13358

**Published:** 2024-10-30

**Authors:** Carlos Rocha Gomes Torres, Talita Portela Pereira, Susanne Effenberger, Alessandra Bühler Borges

**Affiliations:** ^1^ Department of Restorative Dentistry Sao Paulo State University—UNESP, Institute of Science and Technology Sao Paulo Brazil; ^2^ Department of Conservative Dentistry and Periodontology LMU Klinikum Munich Germany

**Keywords:** color masking, esthetic, molar incisor hypomineralization, resin infiltration, transillumination

## Abstract

**Objective:**

This article aims to describe a new technique for predicting the results of resin infiltration procedure in molar incisor hypomineralization lesions, named Infiltration Monitoring by Transillumination. The technique involves the use of transillumination together with ethanol application during the steps of lesion body opening and resin penetration. It provides color contrast that enhances the removal of the less porous surface layer and controls the effectiveness of resin infiltration within the lesion.

**Clinical Considerations:**

The clinical procedure presented illustrates the steps involved in the resin infiltration procedure for color masking of molar incisor hypomineralization lesions in anterior teeth, highlighting the use of transillumination both for monitoring the lesion body opening step and the resin infiltration process.

**Conclusions:**

The monitoring with transillumination during the ethanol test can assist the removal of the enamel external layer over the lesion, necessary to expose the inner porosity to be infiltrated, in a very precise and conservative way. In addition, it can effectively help to determine the moment when the infiltrant resin has fully penetrated the lesion.

**Clinical Significance:**

The Infiltration Monitoring by Transillumination technique offers the possibility to precisely control the infiltration procedure in molar incisor hypomineralization lesions, thereby improving the predictability of the esthetic outcome.

## 1 Introduction

The resin infiltration procedure was originally developed to arrest white spot lesions on the proximal surfaces of posterior teeth, controlling the caries lesion progression by preventing cavitation [1, 2]. However, its ability to change the lesion's optical properties and produce color masking has shifted the focus of the technique [3]. Currently, the main use of the resin infiltration is to promote the esthetic improvement of the teeth. The original procedure developed for caries infiltration is based on three steps: first, the removal of the outer less porous lesion layer using a strong hydrochloric acid gel for 2 min, allowing to access to the porous lesion body; second, complete drying of the tissue using pure ethanol and air blow; and finally, the penetration of resin monomers into the lesion body for 3 min [4, 5, 6]. If the inner part of the lesion is properly accessed, a slight change in the color can be observed during the ethanol application, serving as a preliminary test to predict the masking results of the infiltration. This procedure has been named “ethanol test” [6, 7].

The favorable esthetic results observed in color masking of caries lesions on anterior teeth, such as those caused by biofilm accumulation around fixed orthodontic appliances, have encouraged clinicians to apply the technique for masking developmental defects of the enamel, such as those resulting from fluorosis, trauma or molar incisor hypomineralization (MIH) [8]. However, the distinct histopathological characteristics of each lesion have impaired the predictability of the procedures, and the results were extremely variable [9, 10, 11]. The characteristics of fluorotic lesion provided, on most cases, similar results to the caries lesions infiltration using the same protocol. However, for traumatic lesions and those related to MIH, the conventional approach was mostly disappointing [12].

The predictability of outcomes is influenced by different factors, including the thickness of the hypermineralized surface layer of the lesion, lesion depth, organic content, and the presence of normal intact enamel covering the lesion margins or, in some cases, the whole lesion body [9, 12, 13]. These characteristics interfere with the effectiveness of the procedure's different steps, mainly the etching time and duration of the infiltration [6, 7].

It is important to highlight that the key for the procedure's success is to ensure full access to the lesion body. The thickness of the hypermineralized surface layer can substantially vary [14, 15], and the standard 2‐min etching time developed for caries lesions may not be enough in all cases. Therefore, extended etching times have been proposed [16], as well as the previous surface abrasion using sandblasting or microabrasion with acidic/abrasive paste [7].

The lesion depth impacts the infiltration time, being required in some cases an extended procedure beyond the standard 3 min technique. Some studies suggested to increase the infiltration time to 10, 15, or even 30 min [16]. However, the adoption of a standard protocol may not be adequate, considering the wide variability in lesion morphology.

Regarding margins, fluorotic lesions are quite similar to caries lesions, exhibiting open margins that form an obtuse angle in relation to the surface, allowing adequate resin penetration at the edges [17]. However, in MIH lesions, the margins are generally closed, forming an acute angle with the surface [9, 12]. This impairs the resin penetration at this region, creating a non‐infiltrated halo that jeopardizes the esthetic outcome of the treatment. Additionally, the presence of intact enamel on the MIH lesion surface, with considerable thickness variation, prevents its complete removal using the standard acid etching protocol. Without fully opening of the lesion body, proper masking cannot be obtained. To address this issue, Attal et al. proposed the technique called “deep infiltration” [12]. It was based on the removal of a thin layer from intact enamel surface at center and margins of the lesion, followed by resin infiltration and resin composite restoration of the prepared area [18]. This technique is less invasive when compared to the “macroabrasion technique” used in conventional restorations [19, 20].

Since the characteristics of the lesions determine the clinical protocol, it is evident that the main factor to improve the outcome predictability lies in accurately diagnosing these attributes. To aid in this, the transillumination technique has been used [18, 21]. The procedure is based on the placement of a light source on the lingual side of the teeth to be analyzed. As the light passes through the tooth structure, it is blocked by the lesion body but transmitted through the intact structure. When observed from the labial surface, the lesion area appears dark. The deeper the lesion, the darker its appearance. This method is primarily used before the treatment, for diagnostic purposes only. Marouane et al. proposed that the intact enamel covering the margins of lesion body, which may block the resin penetration in MIH lesions, can be identified by the presence of blurry aspect and diffuse edges, whereas open margins appears as clearly demarcated edges [22, 23, 24]. This characteristic can be properly observed getting a high quality image by a professional photographic camera [22]. To ensure full resin penetration at the margins, the authors suggested to cut the surface with a fine‐grained diamond bur until well defined edges are visible at all margins [22]. However, identifying the characteristics of these margins is challenging for the clinicians unless a high‐resolution image is obtained, transferred to a computer screen, and carefully analyzed. This makes this technique less attractive for routine clinical practice.

In this paper we propose an improved approach to the clinical management for the resin infiltration procedure of MIH lesions, called “Infiltration Monitoring by Transillumination” (IMT). This technique is based on the use of transillumination during two steps of the infiltration procedure: ethanol application and resin infiltration. The simultaneous use of the light source during these steps creates greater color contrast allowing a clear observation, by naked eye, of the ethanol test effectiveness and real time monitoring of resin infiltration, to ensure that a complete penetration of the infiltrant resin has been achieved. To allow the resin infiltration monitoring, a light source that does not promote light curing of the infiltrant resin is required. This can be achieved using a low‐intensity white light source for a short period of time or a yellow/orange light source. The technique is detailed in this paper by a clinical case.

## 2 Case Report

**FIGURE 1:**
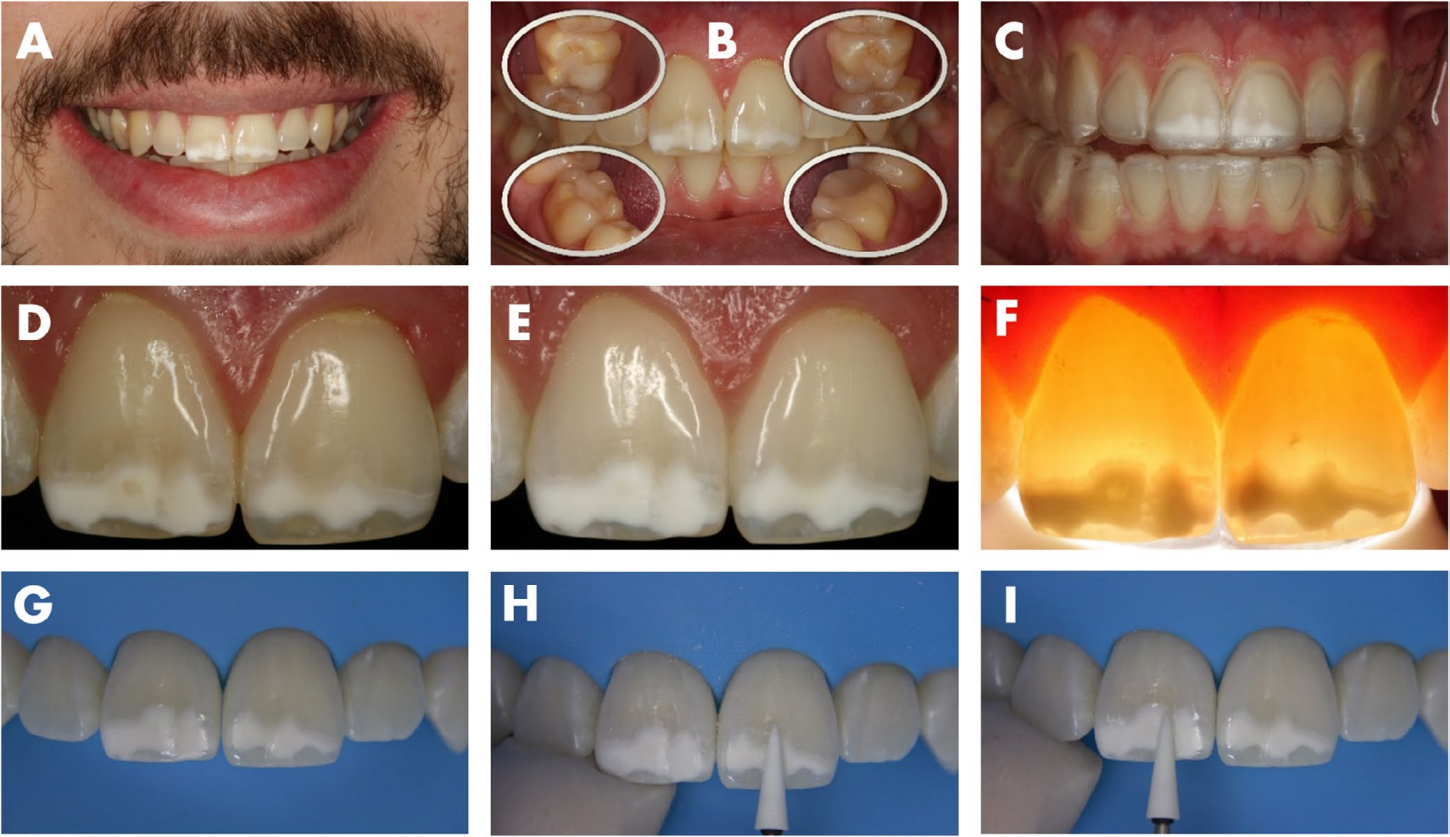
Beginning of the clinical procedure. (A) Baseline aspect of the smile. (B) Lesions in the first molars. (C) Printed bleaching trays placed in dental arches. (D) Central incisors before bleaching. (E) Central incisors after bleaching. (F) Initial transillumination. (G–I) Rubber dam isolation and initial wear of the lesions surface using an abrasive stone.

A 25‐year‐old patient attended the University clinic looking for esthetic improvement of the smile. His main complaint was the presence of two demarcated white lesions in the central incisors (Figure 1A). The clinical examination showed hypomineralized areas on all first molars (Figure 1B), allowing the diagnosis of MIH. During the anamnesis, the patient declared that besides the presence of the white lesions, he was not satisfied with his natural tooth color, which was A3 according to the VITA classical A1‐D4 shade guide (VITA Zahnfabrik, Germany).

The patient's dental arches were scanned, and a bleaching tray was printed (LuxaPrint Orthoflex, DMG, Hamburg, Germany) and tested for fitting in the patient's mount (Figure 1C). Bleaching was performed with 10% carbamide peroxide, 8 h a day for 2 weeks (Opalescence PF, Ultradent, South Jordan, UT, USA). After bleaching, both the sound tooth structure and the lesions appeared whitish (Figure 1D,E). A washout period of 2 weeks was waited before performing the infiltration procedure, allowing enough time for the complete release of hydrogen peroxide and active oxygen species from within the teeth. The initial transillumination was performed using white light from the diagnostic tip of a light‐curing unit (Radii Xpert, SDI, Australia), allowing to assess lesion depth and to analyze the margins (Figure 1F).

The infiltration procedure was recorded using a Video Recording Camcorder (Handycam 4K, Sony, Japan) attached to the dental chair. After rubber dam isolation, an aluminum oxide finishing abrasive stone (Dura‐White Stones, Shofu, Kyoto, Japan) in a low‐speed handpiece was used to gently remove a thin layer of the hypermineralized surface of the lesion (Figure 1G–I).

**FIGURE 2:**
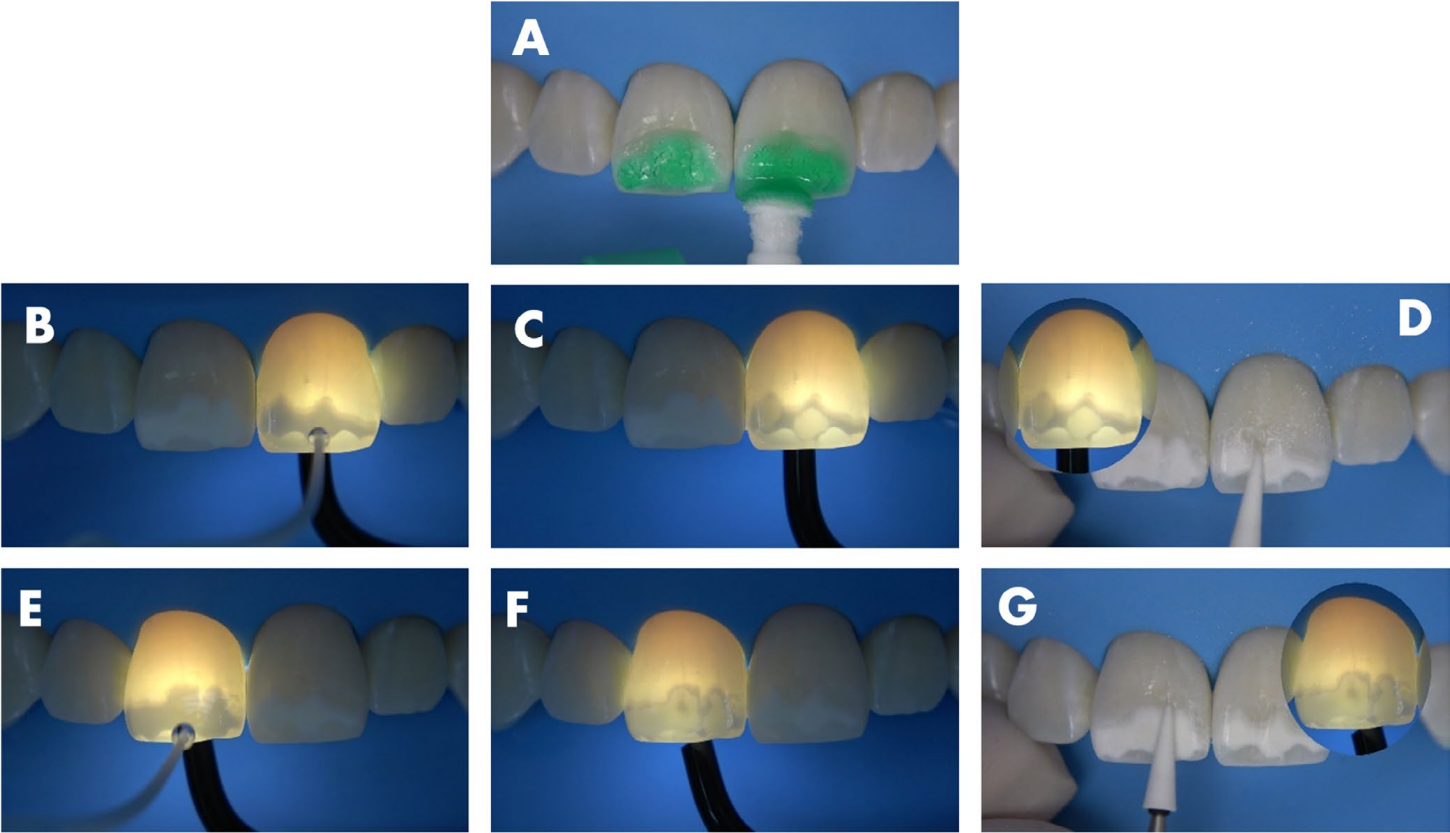
(A) First acid etching for 10 s. (B and C/E and F) Ethanol application and monitoring under transillumination allowing an easy detection of the opened areas of the lesion body by the clear aspect, while the dark areas indicates that the lesion body remains close. (D and G) Precise wear of the dark margins using the tip of the abrasive stone.

**FIGURE 3:**
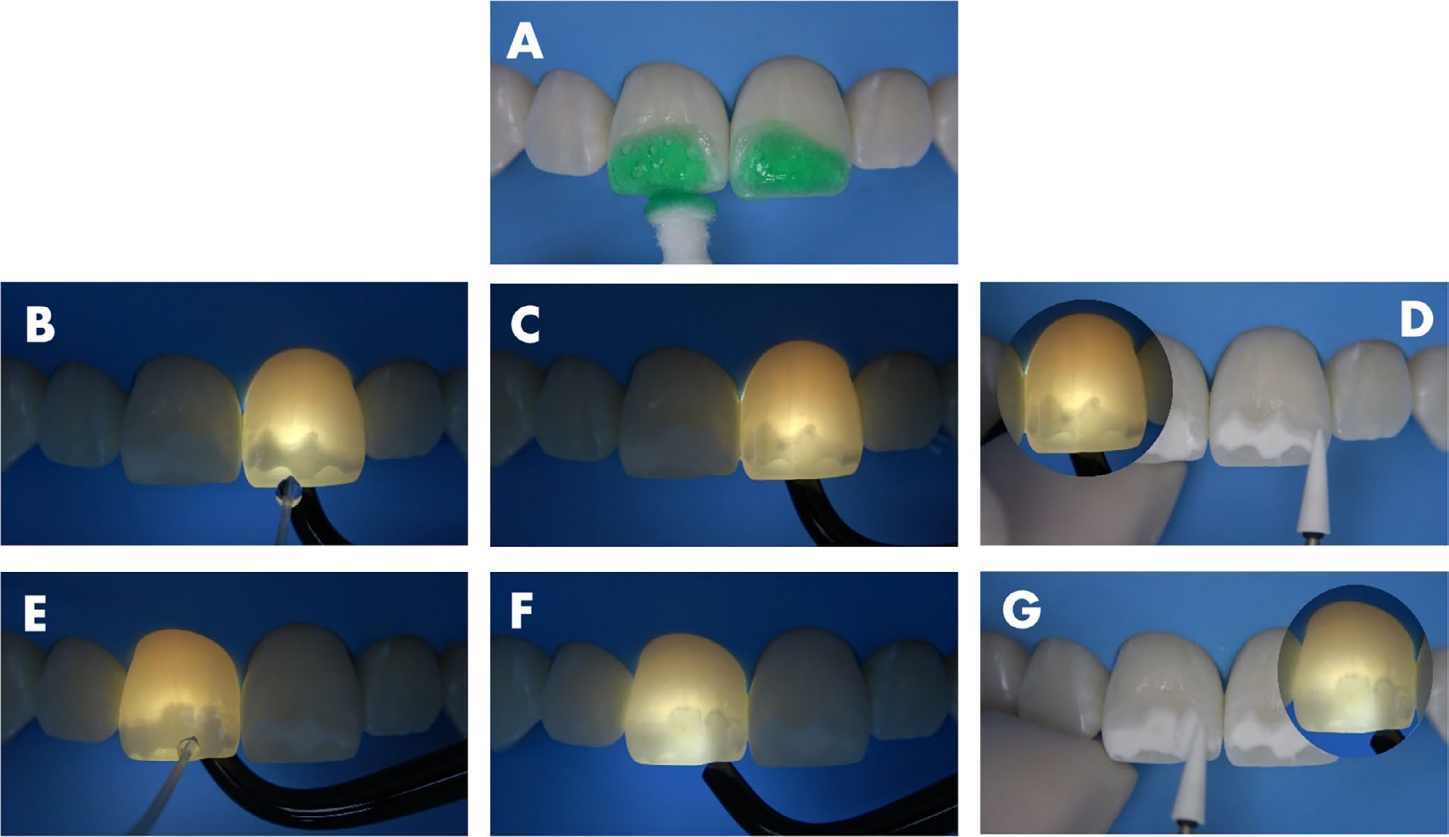
(A) Second acid etching for 10 s. (B and C, E and F) Ethanol application and monitoring under transillumination allowing the detection of the opened and still closed areas of the lesions. (D and G) Precise abrasion in the dark areas.

**FIGURE 4:**
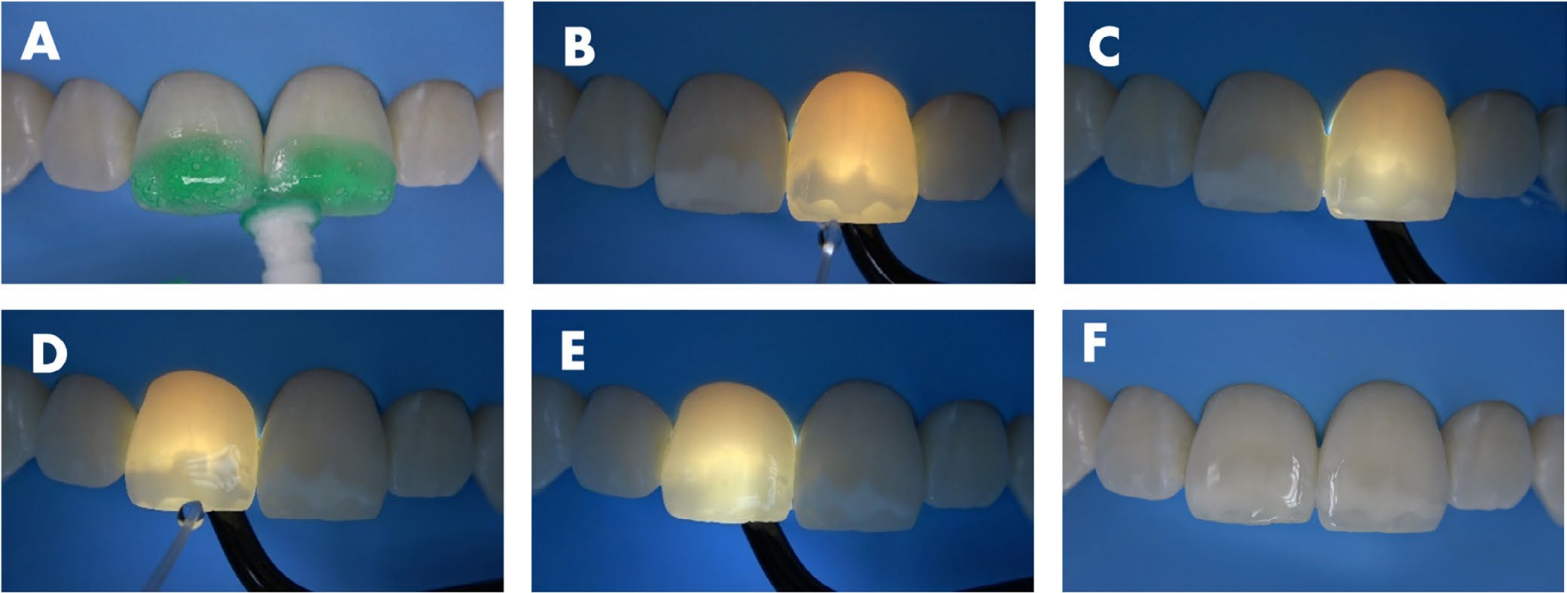
(A) Last acid etching for 10 s. (B and D) Ethanol application under transillumination. (C and E) In both teeth the whole lesions were clear under transillumination, indicating that the lesion bodies were fully opened. (F) Clinical appearance of the lesions after ethanol application without transillumination.

The surface was then etched with the 15% hydrochloric acid gel (Icon Etch, DMG) for 10 s to remove the smear layer created by the abrasion (Figure 2A). After that, a white light transillumination device (Microlux, Addent, Connecticut, USA) was placed on the lingual surface of the left central incisor while pure ethanol (Icon Dry, DMG) was applied to the lesion surface, and the effect was monitored. Over time, the area where the ethanol was capable of penetrating became easily visible to the naked eye, by changing the aspect from dark to light (Figure 2B,C), indicating that in this region the lesion body was accessible. Once the penetration stabilized, a picture was taken with a cell phone camera to register the areas where the lesion remained closed (dark regions), preventing ethanol penetration. The procedure was repeated on the right central incisor (Figure 2E,F). Then, the aluminum oxide stone was used again only in the dark areas (Figure 2D,G). Afterward, a 10 s acid etching was repeated followed by another round of transillumination and ethanol application as described, showing the reduction of the still closed areas (Figure 3A–C,E,F). The abrasion procedure was repeated once more, targeting only the remaining dark areas (Figure 3D,G). After that, the acid etching was repeated and the ethanol penetration monitored under transillumination, showing a complete opening of both lesions (Figure 4). This process allowed for easy and continuous monitoring of the effectiveness of the lesion body opening procedure. Figure 4F shows the clinical appearance of the lesions after ethanol application without transillumination, highlighting how difficult is do determine by naked eye the lesion opening status without the aid of an extra light source.

**FIGURE 5:**
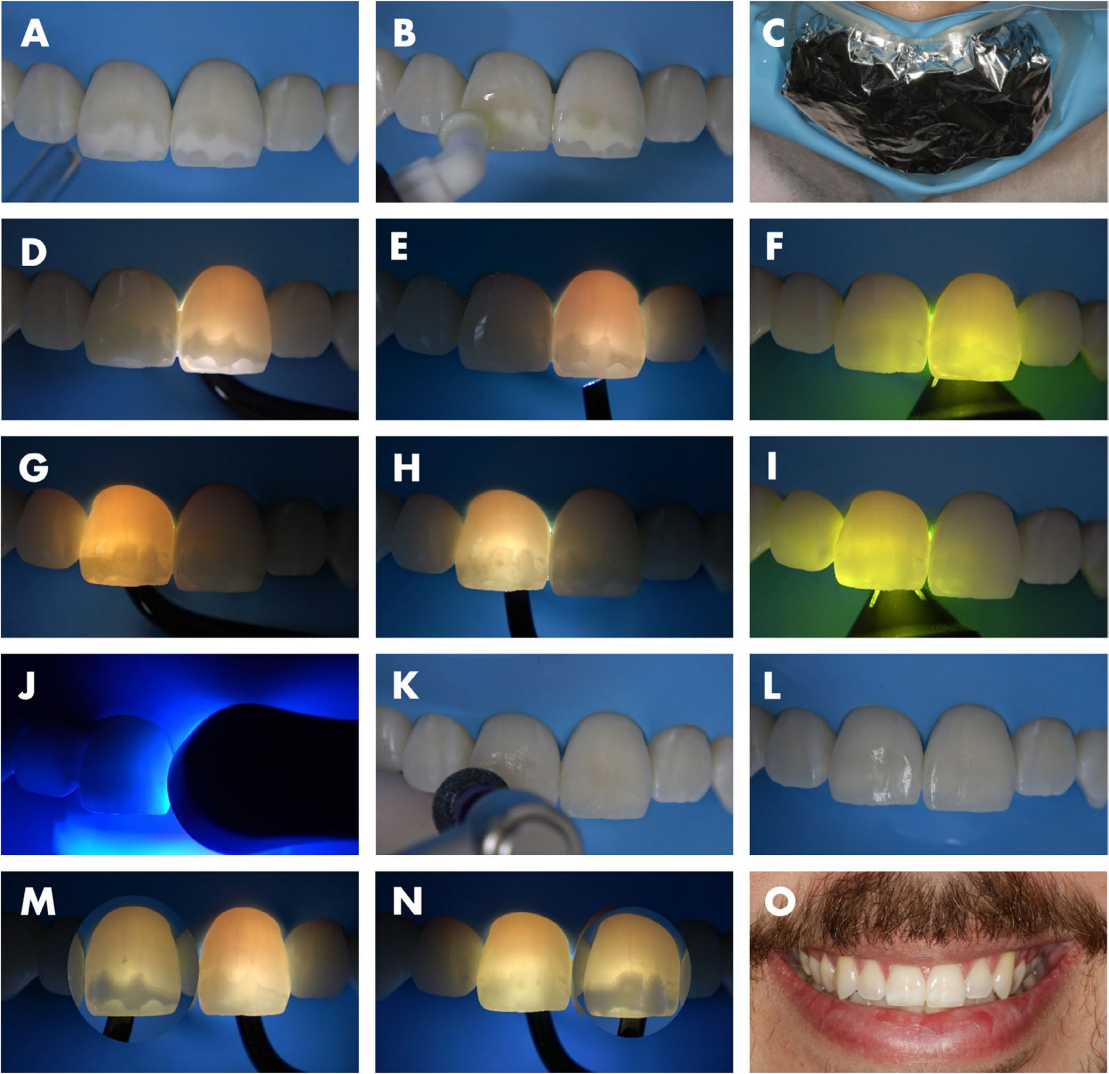
(A) Drying with air blast. (B) Resin infiltrant application. (C) Protection against the environmental light during infiltration. (D–I) Resin infiltration monitoring under transillumination. (J) Light curing. (K) Polishing. (L) Immediate result. (M and N) Comparison of the lesions under transillumination before and after resin infiltration. (O) Final result after 7 days.

The surface was then air dried, and the infiltrant resin (Icon Infiltrant, DMG) was applied (Figure 5A,B). The teeth were protected from the environmental light using an aluminum foil to prevent premature curing of the infiltrant resin (Figure 5C). From time to time the infiltration process was monitored using a white light source (Figure 5D,E,G,H) or orange light source (TransLume lens—Valo light‐curing unit, Ultradent, USA) (Figure 5F,I). When a fully clear image of the lesion was visible, with no dark areas, a complete infiltration was achieved (Figure 5F,I). The excess of infiltrant was aspirated with a thin oral suction cannula, and the proximal area cleaned with dental floss. Light curing was performed (Figure 5J), followed by another application of the infiltrant for 1 min. The excess was removed, and light curing was repeated. The surface was polished with abrasive discs (Super‐Snap, Shofu, Kyoto, Japan) and silicon carbide abrasive brush (Occlubrush, Kerr, Orange, CA, USA) (Figure 5K). In Figure 5M,N, it is possible to compare the baseline and the final aspect of the lesions under transillumination.

The immediate and the final conditions are presented in Figure 5L,O. The prominence of the whitish lesion was significantly reduced, indicating an effective masking effect. The patient expressed satisfaction with the esthetic result.

## 3 Discussion

The resin infiltration procedure of MIH lesions is challenging due to the varying depth of the lesions, their high organic content, and their unique morphology, which often exhibits an intact enamel surface layer extending across the entire lesion or only at its margins [9]. If not properly removed, this intact enamel can hinder the infiltration of the low‐viscosity resin inside the lesion body, resulting in incomplete penetration and the presence of an opaque halo around the centrally infiltrated lesion, which compromises effective masking [12].

Previous attempt to use the clinically visible characteristics of the lesion that could improve the prediction of the infiltration procedure had failed [13, 25]. Up to now the transillumination during the diagnosis session was the best approach to proper analyze the lesion depth and estimate the infiltration results [22]. It may help to determine the presence of diffuse margins where it is expected to have defective penetration of the infiltrant resin. The original technique describe by Marouane et al. suggest that the diffuse margins should be abraded with a bur up to convert them to demarcated edges, before starting the infiltration procedure [22]. However, this approach requires a skilled observation of the lesion, preferably with high quality clinical images under magnification on a computer screen. Although recognizing the viability of the methods, many clinicians, even those with large professional experience, expressed difficulties do determine whether the lesions margins were diffuse or demarcated. However, with the newly proposed IMT technique, the use of ethanol under transillumination provides high contrast and a clear vision, even to the naked eye, of the opening of the lesion body. This simplifies the clinical procedure for accessing the lesion, even for less skilled dentists, which is crucial to the success of the infiltration procedure.

Regarding resin penetration, considering the great variability in lesion depth, as well as its porosity and penetration speed, it is challenging to stablish an ideal resin application time for achieving adequate infiltration [10, 14]. This can be correlated to the presence of a high organic content and reduced surface free energy [26]. While in a normal mature enamel the organic content is just around 4%, in MIH lesion it can reach up to 68% [27]. In an attempt to solve this problem, some studies proposed the previous application of a deproteinizing agent, such as sodium hypochlorite solution [10, 28, 29]. However, this approach does not lead to a real success, as it remains challenging to actually determine the best infiltration protocol. Thus, instead recommending a pre‐established infiltration time, the IMT technique allows clinicians to clearly identify the exact moment when the lesion infiltration has reached its maximum, indicated by the clear aspect of the lesion, as shown in (Figure 5F,I).

In the IMT technique, the etching time was also reduced. In the original technique developed for caries infiltration, the 15% hydrochloric acid gel step was created by the manufacturer to remove the less porous hypermineralized surface layer of approximately 45 μm in clinically reasonable time [5]. However, for MIH lesions, some studies described much thicker intact surface layer, up to 300 μm [14, 15]. Therefore, multiple etching procedures have been used by the clinicians in an attempt to open the lesion body, which may eventually create a concavity on the tooth surface due to the faster dissolution of the lesion's central area. The etching procedure has been repeated by the dentists without knowing exactly when to stop and even so, the procedure outcome has been sometimes unsuccessful, resulting in a non‐infiltrated marginal halo. This way, the direct removal of a thin layer of lesion surface with a fine bur, as proposed by Marouane et al. [22], seems to be the best approach, and this was performed in the clinical case described (Figure 3D,G). However, in the IMT technique, the detection of the closed lesion areas can be easily accomplished, allowing for the precise reapplication of the abrasive rotary instrument in these areas. This approach reduces the amount of enamel removed to the minimum necessary and shortens the etching procedure to only 10 s, with the unique purpose of removing the smear layer created by the abrasive instrument. This is feasible because ethanol penetration serves as a marker for successful access to the lesion body.

As demonstrated in the clinical case description, the IMT technique can reduce treatment time by objectively determining the successful opening of the lesion body, save material by reducing the acid gel consumption, advise the dentist on when the infiltration has reached its maximum, and enhance the esthetic predictability and success of the infiltration procedure. However, clinical studies are recommended to scientifically validate that this approach can significantly improve the quality of the infiltration procedure for MIH lesions, involving a larger number of patients and a variety of lesion types. Additional studies are also necessary to quantify and scientifically compare tissue removal using the traditional approach in relation to the IMT technique.

## 4 Conclusion

It was concluded that the IMT technique enhances the predictability and success of esthetic treatment for MIH lesions by optimizing and guiding the procedures. It helps control the amount of enamel removal required for effective resin infiltration, indicates when the lesion body has been properly opened, and monitors when the infiltration procedure has reached its maximum.

## Disclosure

The authors do not have any financial interest in the companies whose materials are included in this article. SE is an employee of DMG Dental‐Material Gesellschaft mbH, the company that is marketing the commercial resin infiltrate Icon, but they do not receive any personal benefits from the sale of this product. SE holds a part‐time position of the Department of Conservative Dentistry and Periodontology, Ludwig‐Maximilians University Munich, Germany.

## Conflicts of Interest

The authors declare no conflicts of interest.

## Data Availability

Research data not shared.
